# *Clostridium difficile* in faeces from healthy dogs and dogs with diarrhea

**DOI:** 10.1186/1751-0147-55-23

**Published:** 2013-03-12

**Authors:** Karl-Johan Wetterwik, Gunilla Trowald-Wigh, Lise-Lotte Fernström, Karel Krovacek

**Affiliations:** 1 Department of Biomedical Sciences and Veterinary Public Health, Division of Bacteriology and Food Safety, Swedish University of Agricultural Sciences, P.O. Box 7028, Uppsala, SE-750 07, Sweden

**Keywords:** Clostridium difficile, Dog, Faeces, Toxins, Ribotyping, Antibiotic resistance pattern

## Abstract

**Background:**

This study was conducted to evaluate the faecal occurrence and characterization of *Clostridium difficile* in clinically healthy dogs (N = 50) and in dogs with diarrhea (N = 20) in the Stockholm-Uppsala region of Sweden.

**Findings:**

*Clostridium difficile* was isolated from 2/50 healthy dogs and from 2/20 diarrheic dogs. Isolates from healthy dogs were negative for toxin A and B and for the *tcdA* and *tcdB* genes. Both isolates from diarrheic dogs were positive for toxin B and for the *tcdA* and *tcdB* genes. The *C. difficile* isolates from healthy dogs had PCR ribotype 009 (SE-type 6) and 010 (SE-type 3) whereas both isolates from dogs with diarrhoea had the toxigenic ribotype 014 (SE-type 21). One of the isolates from healthy dogs was initially resistant to metronidazole.

**Conclusions:**

This study revealed presence of toxigenic *C*. *difficile* in faecal samples of diarrheic dogs and low number of non- toxigenic isolates in healthy dogs from Uppsala-Stockholm region in Sweden. However, more comprehensive studies are warranted to investigate the role of *C. difficile* in gastrointestinal disease in dogs.

## Background

The role of *C.difficile* in gastrointestinal disease in dogs is not yet defined. Moreover, interspecies transmission of *C. difficile* has also been suggested [1]. The virulence of *C. difficile* is mainly related to the presence of two exotoxins, A and B that are encoded by *tcd*A and *tcd*B genes, respectively [2]. In addition, some strains express a binary toxin (CDT) [3].

The aim of the present study was to evaluate the presence of *C. difficile* in faeces from healthy and diarrheic dogs in the Stockholm-Uppsala region, Sweden. Toxin production, ribotyping and antibiotic resistance pattern of the isolates was also revealed.

## Material and methods

### Animals and sampling

Faecal samples from 50 healthy, randomly selected, and 20 diarrheic dogs (8 months-12 years old, median age 5 years) of varying breeds and from different households were investigated. Eighteen of the diarrheic dogs were hospitalized due to acute gastrointestinal disease involving diarrhea, whereas the remaining two dogs were outpatients and sampled at veterinary clinics. Use of systemic antibiotics within the last three months was recorded in a questionnaire for the healthy dogs. Samples were collected directly upon defecation. To prevent contamination, 10–20 g of faeces was collected with a spoon and stored in a sterile plastic sampling tube. The samples were kept refrigerated, and processed in the laboratory within 12 h after collection.

### Culture conditions and bacteriological analysis

Faecal samples were plated onto *C*. *difficile* agar (CM0601 Oxoid, Basingstoke, UK) and used to inoculate *C. difficile* enrichment broth. *Clostridium difficile* agar was supplemented with moxalactam and norfloxacin (SR0173E Oxoid) and 5% horse blood (National Veterinary Institute, SVA, Uppsala, Sweden), and incubated anaerobically for 48 h at 37°C. *Clostridium difficile* broth was prepared with the ingredients for CM0601 (agar excluded), supplemented with moxalactam and norfloxacin. Briefly, 5 g of each faecal sample was added to 10 mL broth and mixed by vortex and incubated anaerobically at 37°C for 10 days. After incubation, the suspension was streaked onto *C*. *difficile* agar and Fastidious Anaerob Agar (FAA agar, SVA, Uppsala, Sweden) containing 5% horse blood and incubated for 48 h.

### Identification of the isolates

*Clostridium difficile* was tentatively identified on the basis of Gram stain and morphology, characteristic horse manure odour, L-proline aminopeptidase activity (Oxoid), and then confirmed by the latex agglutination test (*C. difficile* test kit DR1107A, Oxoid).

### Toxin detection of *C. difficile*

Toxin A detection was performed on fresh faeces, within 12 h after sampling, using the *C. difficile* toxin A test (TD 0970A, Oxoid Ltd). Positive control (*C. difficile* strain ATCC 43255) and negative control were included in each experiment, according to the instructions of the manufacturer. Detection of toxin B was performed by inoculation of isolates into *C. difficile* broth. The sterile culture filtrates (48 h incubation at 37°C under anaerobic condition) were used in the Vero cell assay as previously described [4].

### Detection of *C. difficile* toxin A and toxin B genes

Presence or absence of toxin A and toxin B genes were evaluated by using PCR amplification of the A3 and B1 fragments using primer pairs and PCR protocol described by Rupnik [5]. The analysis was performed on *C. difficile* isolates.

### PCR ribotyping of *C. difficile*

PCR ribotyping was performed according to Stubbs et al. [6] with modifications described by Svenungsson et al. [7]. The type was given according to the international nomenclature by using the ribotype nomenclature of the Cardiff Anaerobe Reference Laboratory (Cardiff, Wales, UK), or when reference isolates were missing, as SE-type (Swedish type).

### Antibiotic resistance pattern of the isolates

*Clostridium difficile* isolates were analysed for their susceptibility to five different antibiotics (Table 1). Antimicrobial susceptibility patterns and MIC values were determined by the Etest (AB Biodisk, Solna, Sweden) according to the manufacturer’s instructions. The breakpoints were chosen according to The European Committee on Antimicrobial Susceptibility Testing (EUCAST) [8]. For quality control a clinical isolate namely, (CD0903), which is sensitive to the antibiotics in the test, was used in parallel for each incubation. The MICs were below current breakpoints and no drift over time has been detected (the measured MIC ranges (mg/L) for the control isolate are: erythromycin 0.25-1; clindamycin 0.25-1; moxifloxacin 0.5-2; metronidazole: 0.032-0.125; vancomycin: 0.25-0.5).

**Table 1:** **Ribotypes, tcdA/tcdB and antibiotic susceptibility patterns with MIC values (mg/L) of *C. difficile *isolated from dogs in Sweden**

**Isolate no./group**	**Ribotype**	***tcd*A &*tcd*B**	**Metronidazole**	**Vancomycin**	**Clindamycin**	**Erytromycin**	**Moxifloxacin**
35/healthy	009	-	64,0*	0,5	0,5	1,0	1,0
38/healthy	010	-	0,064	0,5	2,0	2,0	1,0
56/diarrheic	014	+	0,125	0,5	2,0	2,0	2,0
61/diarrheic	014	+	0,032	0,5	4,0	1,0	2,0

* Result from the initial test.

## Results

*Clostridium difficile* was isolated from 2 of the 50 healthy dogs and from 2 of the 20 diarrheic dogs investigated. *Clostridium difficile* was isolated both from primary culture and from the enrichment broth after 10 days of incubation, except the isolate no. 56 that was recovered only after enrichment.

### Toxin production of *C. difficile*

Both *C. difficile* isolates (no. 56 and 61) from diarrheic dogs were toxin B producers, and were positive for the *tcd*A and *tcd*B genes (Table 1). However, samples from both dogs were negative on the initial *C. difficile* toxin A test performed on fresh faeces. Faeces from 4 additional diarrheic dogs tested positive for toxin A test but bacteria were not isolated from these samples. The *C. difficile* isolates (no. 35 and 38) from healthy dogs were negative for the *tcd*A and *tcd*B genes (Table 1). Culture filtrates from these samples were negative for toxin B, and all faeces from the animals from which the isolates were obtained were negative for toxin A.

### PCR ribotyping

The isolates from healthy dogs were classified as PCR ribotype 009/SE-type 6 (isolate no. 35) and 010/SE-type 3 (isolate no. 38), whereas both isolates from diarrheic dogs had PCR ribotype 014/ SE-type 21 (Table 1).

### Antibiotic treatment of the dogs and resistance pattern of the isolates

Two of the healthy dogs investigated were treated with metronidazole and amoxicillin with clavulanic acid respectively, within three months before sampling. The isolate no. 35 was isolated from the dog that was treated with metronidazole, and this isolate was initially resistant to high levels of metronidazole: 64 mg/L (Table 1). However, high level metronidazole resistance was not maintained; subsequent subcultures following recovery from frozen storage were resistant to between 2 and 8 mg/L of antibiotic. Isolate no. 38 was isolated from a dog without history of antibiotic treatment, and this strain did not show elevated resistance to the antibiotics tested. Seven of the 20 diarrheic dogs were treated with antibiotics at the animal hospital or within 3 months before sampling. Antibiotic treatments in these dogs included amoxicillin (N = 1), ampicillin (N = 2) and metronidazole (N = 2), metronidazole and amoxillin with clavulanic acid (N = 1), amoxicillin, ampicillin and metronidazole (N = 1). Isolate no. 56 was from a dog treated with metronidazole during hospital stay. Isolate no. 61 was isolated from a dog treated (at the hospital), during recurrent diarrheic episodes, with ampicillin, metronidazole and amoxicillin respectively. One of the dogs that tested positive for the presence of toxin A, but was culture negative, had been treated with metronidazole and amoxicillin with clavulanic acid. Resistance patterns of the isolates are shown in Table 1.

## Discussion

*Clostridium difficile* was isolated in 2/50 of the healthy dogs and in 2/20 of the diarrheic dogs. Additionally, toxin A was detected in the faeces of diarrheic dogs and toxigenic bacteria were only isolated from diarrheic dogs, whereas toxin A was not detected in the faeces of any healthy dogs. Both isolates from healthy dogs were PCR negative for the *tcd*A and *tcd*B toxin genes. In four diarrheic dogs toxin A was detected in faeces samples but *C. difficile* was not isolated. On the other hand the *C. difficile* isolated from faeces of two diarrheic dogs were cdtA/cdtB positive but negative in the initial toxin A test performed on fresh faeces. Possible explanations for this phenomenon could be that *C. difficile* is a strict anaerobe bacterium and the recovery and cultivation of the microorganism can be difficult. Also, the bacteria can be present in the faeces although in very small number and therefore, in some cases, undetectable. Furthermore, very little is known about the actual excretion of toxins in the different phases of *C. difficile* colonisation/infection. This factor could also explain the negative results in the initial toxin A test.

There is wide variation in the reported *C. difficile* carriage rate among household pets. Different studies describes occurrence of *C. difficile* in faecal samples of dogs ranging from 0% to 21% [9-12]. Both toxigenic and non-toxigenic isolates are reported*.* Several factors may contribute to these discrepancies, including different methods of isolation and detection, prior use of antibiotics, geographical location, age or breed of animals.

Both isolates from diarrheic dogs had the toxigenic ribotype 014 associated with clinical disease in humans [13], while isolates from healthy dogs revealed nontoxigenic PCR ribotypes 009 and 010. Ribotype 010 is occasionally isolated from human faeces in Sweden while type 009 is uncommon [14], whereas ribotype 014 is commonly detected in clinical specimens from human patients in Sweden [15].

Evidence of antibiotic resistance in *C. difficile* isolates from humans is growing, but reports of antibiotic resistant strains from dogs are rare [13,16]. Marks and Kather [16] reported that all *C. difficile* isolated from diarrheic and nondiarrheic dogs were susceptible to metronidazole and vancomycin, leading to the recommendation that these drugs are appropriate for use in treating infection. However, in our study, one of the *C. difficile* isolates from a healthy dog (no. 35), treated with metronidazole, was initially resistant to metronidazole (MIC = 64 mg/L). The dog from which this isolate originated was treated for gingivitis with metronidazole one month before sampling. The high level of metronidazole resistance in this purified strain was unstable and decreased to 2 mg/L, which may be due to the absence of continual antibiotic selection. This phenomenon has been reported by Pelaez et al. [17] and Huang et al. [18] on human isolates and is not fully understood. The lack of continuous selection in vitro might lead to loss of resistance. Genetic determinants of resistance in *C. difficile* are not known but may be on an unstable plasmid that is lost without continuous selection. Metronidazole unresponsive *C. difficile* infection has been discussed as an emerging problem in humans [19] but resistance against metronidazole has not to our knowledge previously been reported in *C. difficile* isolates from dogs.

In conclusion, our work contributes to the growing evidence on the occurrence of toxigenic and nontoxigenic *C. difficile* in dogs. This study revealed presence of toxigenic *C*. *difficile* in faecal samples of diarrheic dogs and low number of non- toxigenic isolates in healthy dogs. However, more comprehensive studies are warranted to investigate the role of *C. difficile* in gastrointestinal disease in dogs. Also, the finding of toxigenic ribotype 014 in diarrheic dogs might imply a potential zoonotic risk. In addition, one *C. difficile* isolate was initially resistant to metronidazole, and acquisition of resistance to this common drug of choice in dogs should be considered in veterinary clinical practice.

## Competing interests

The authors declare that they have no competing interests.

## Authors’ contributions

KK was responsible for the study design, organisation of the work, analyses of the data and preparation of the manuscript. KJW carried out the sampling, identification and toxin detection of the isolates, participated in organisation of the work and in the data analyses. LLF was responsible for the technical assistance during the course of the study. GTW was co-responsible for the study design, organisation of the work and analysis of the data and preparation of the manuscript. All authors have contributed to the writing of the manuscript, and read and approved the final version of the manuscript.
